# Effects of different planes of milk feeding and milk total solids concentration on growth, ruminal fermentation, health, and behavior of late weaned dairy calves during summer

**DOI:** 10.1186/s40104-021-00615-w

**Published:** 2021-09-02

**Authors:** R. Shiasi Sardoabi, M. Alikhani, F. Hashemzadeh, M. Khorvash, M. Mirzaei, J. K. Drackley

**Affiliations:** 1 Department of Animal Science, College of Agriculture, Isfahan University of Technology, 84156-83111 Isfahan, Iran; 2 Department of Animal Science, Faculty of Agriculture and Natural Resources, Arak University, 38156-88349 Arak, Iran; 3 Department of Animal Sciences, University of Illinois, Urbana, 61801 USA

**Keywords:** Dairy calf, Milk feeding level, Pre-weaning, Total solids

## Abstract

**Background:**

In recent years, there has been an increasing interest in using high quantities of milk or milk replacer (MR) in heat-stressed calves to alleviate the negative effects of high environmental temperatures on their performance. However, observations have indicated a decline in growth performance in the weaning and post-weaning period, which might be optimized with increasing total solids (TS) in milk and weaning age. This study aims to optimize the effects of higher quantities of milk on late weaned calves' performance by increasing TS concentration or delivery route in summer conditions.

**Method:**

Forty-eight newborn Holstein calves were used in a 2 × 2 factorial arrangement with the factors of pre-weaning total plane of milk (PM) intake (low vs. high) and milk TS content (12% vs. 17%). The treatments were (1) low PM (LPM) intake with 12% TS (TS intake = 45.9 kg), (2) LPM intake with 17% TS (TS intake = 65.1 kg), (3) high PM (HPM) intake with 12% TS (TS intake = 63.7 kg); and (4) HPM intake with 17% TS (TS intake = 90.3 kg). Calves were weaned at d 83, and the study was terminated at d 103 of age. Performance data (every 10 day), skeletal growth (d 80 and 100), ruminal fermentation parameters (d 48 and 91), and behavioral measurements (d 69, 70, 93 and 94) were analyzed as repeated measurements with PROC MIXED of SAS 9.4 (SAS Institute Inc., Cary, NC).

**Results:**

Calves receiving HPM consumed less PMR from d 44 to 83 of age, but they had higher ADG from d 24 to 53 of age compared to those fed LPM (PM × age, *P* < 0.001). In addition, calves receiving milk with 17% TS had lower PMR intake from d 14 to 83 of age, but greater ADG from d 34 to 53 compared to those receiving milk with 12% TS (TS × age, *P* < 0.001). Calves that received HPM had greater skeletal growth parameters compared to LPM-fed calves, with a similar effect evident for calves fed milk with 17% TS compared with those fed milk with 12% TS. Calves receiving milk with 17% TS had greater fecal scores and diarrhea occurrence than those fed milk with 12% TS in HPM, but not LPM.

**Conclusions:**

Increasing PM and milk TS concentration improved growth in summer-exposed calves as demonstrated by increased pre-weaning ADG, pre- and post-weaning BW, and structural growth.

## Background

Environmentally heat-loaded conditions in the dairy industry are a multifactorial problem. Animals activate a variety of physiological, endocrine, and behavioral mechanisms to cope with hot weather conditions. Previous studies have reported that pre-weaning calves raised during summer conditions have reduced comfort, growth rate, and immune function along with greater susceptibility to diseases due to reduced feed intake [1–4]. Recently, it has been reported that decreased starter diet intake in summer months is mostly responsible for impaired growth performance of growing calves, which consequently impairs their future productive performance [5, 6]. In addition, summer conditions increase the maintenance energy requirement for body temperature regulation and reduce the amount of energy available for growth [7]. Besides other management factors for mitigating heat stress [8], nutritional strategies for enhancing nutrient intake during hot weather conditions might be an effective option for enhancing the wellbeing and performance of calves.

During the pre-weaning period, sufficient milk or MR provision is a prerequisite to prevent calf growth depression during summer [3]. Moreover, a great deal of research indicates that enhanced liquid feeding programs during the first weeks of life promote body and skeletal growth and wellbeing compared to conventional limit feeding programs [9–11]. However, despite its beneficial effects, intensive milk feeding programs delay starter diet intake, which may impair rumen development and functionality during weaning as well as post-weaning growth performance [12, 13].

More recently, in a meta-analysis study, it was reported that feeding a higher plane of MR increased ADG and structural growth in the pre-weaning period (d 0–56) but decreased post-weaning growth performance (d 56–112), which could be related to reductions in nutrient digestibility as a result of feeding more MR [14]. Previous studies showed that step-up/step-down milk feeding procedures and later weaning age are ways to overcome the negative effects of providing greater volumes of liquid feed and to promote successful transition from milk to solid feed [15–17]. One study by Hill et al. [18] showed that gradually weaning over a longer period in calves fed greater amount of MR increased digestion and growth in post-weaning period. In addition to greater growth rates, later weaning ages in calves fed high plane of MR resulted in more gut development [19, 20] and reduced signs of stress [16] during weaning. However, responses of calves to nutritional strategies may differ during summer conditions.

Recently, Orellana Rivas et al. [3] hypothesized that offering greater amounts of MR to calves weaned at 49 d of age would enhance dietary energy available for growth during summer. These researchers reported that increasing the amounts of MR feeding from 0.55 to 0.66 and 0.77 kg/d increased BW gain in early-weaned calves. However, further increasing MR intake did not improve growth, perhaps due to large meal sizes and heat stress, which increased incidence of abomasal bloating. Hill et al. [15] reported that later weaning age in calves fed 0.66 kg of DM from MR could overcome some of the ADG lost without influencing calf starter intake during warm summer months in young dairy calves. To our knowledge, however, few studies have investigated the effects of feeding higher planes of milk to late weaned calves during summer.

Although increasing PM intake frequently decreases starter diet intake [14, 21, 22] and apparent nutrient digestibility [23, 24], Azevedo et al. [25] reported that feeding calves with increasing concentrations of total solids (TS) in milk up to 20.4% increased weight gain and skeletal development without compromising starter diet intake during pre- and post-weaning periods. An increase in the amount of solids in the MR during hot weather conditions could guarantee ingestion of the desired amount of nutrients, thus increasing nutritional status, particularly in the first week of life when calves have limited thermoregulatory ability and disease incidence is higher [22]. However, few studies have been conducted on the effects of offering greater amounts of milk TS (up to 1.360 kg/d) to late weaned calves during summer.

This study aimed to investigate the effects of increasing milk TS concentration (17% vs. 12%) and the PM provision (5 vs. 8 kg/d) on performance, rumen fermentation, health, and behavior of late weaned calves during summer. Our hypothesis was that milk with higher TS concentration would promote greater starter intake and enhance performance responses compared to calves receiving greater PM with normal TS concentration. Through this study, we compared the performance and health of late weaned calves receiving equal quantities of milk TS with different delivery routes, including greater PM or milk TS concentration in summer conditions.

## Materials and methods

### Animals, management, and experimental treatments

The study was conducted at the facilities of a local dairy farm (FKA Animal Husbandry and Agriculture Co., Isfahan, Iran) from June to September 2018. All animal procedures were according to the guidelines of the Iranian Council of Animal Care [26]. A total of 48 Holstein calves (40.2 ± 1.54 kg of BW) were separated from their dams immediately after birth, weighted and randomly transferred to individual pens (2.9 m × 1.1 m × 1.8 m; length × width × height) bedded with sand that was cleaned every 3 d by removing all bedding and renewing it with fresh sand. To keep the pens dry and clean, manure was removed daily as needed. All calves were fed 6 L of high quality colostrum (Brix values ≥ 22%; [27]) using nipple bottles after birth and 12 h after the first feeding. Only calves having a serum protein level > 6 g/ dL were included in the study. After colostrum feeding, the calves were fed 4 L of transition milk per day, until the end of the third day. From d 4 onward, non-salable pasteurized milk containing 3.20% ± 0.15% fat, 3.11% ± 0.08% CP, 5.21% ± 0.09% lactose, and 12.0% ± 0.15% TS was warmed to 39 ± 1.0 °C using a water bath and provided to calves in steel buckets individually in 2 meals of equal volumes at 0800 and 1500 h per day until weaning.

**Fig. 1:**
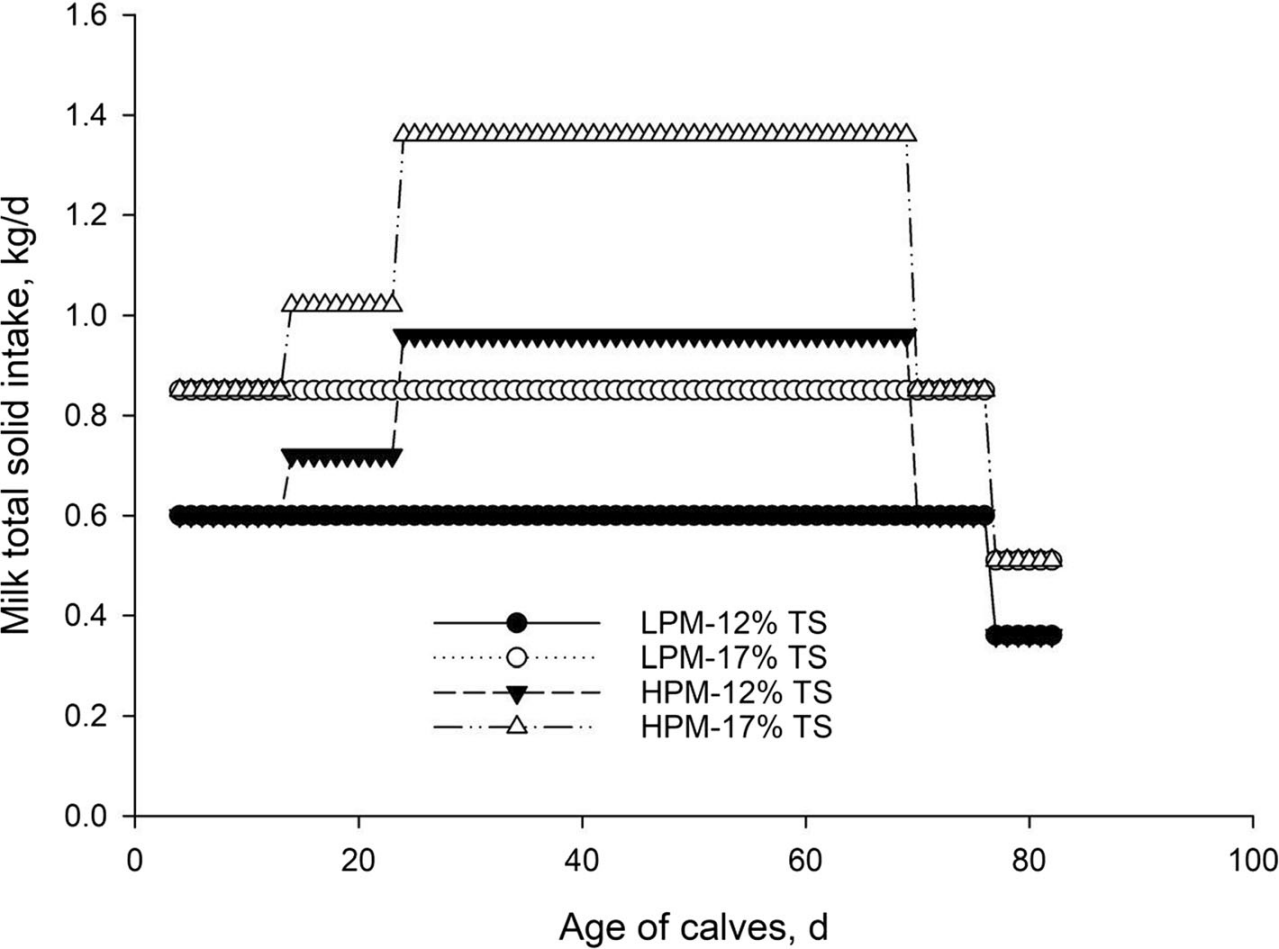
Milk total solids intakes for different feeding treatments during preweaning period. (1) LPM-12% TS (5 L/d milk from d 4 to 76 and 3 L/d of milk from d 77 to 83 of age; total milk intake = 383 L, total milk solids intake = 45.9 kg), (2) LPM-17% TS (5 L/d milk from d 4 to 76 and 3 L/d of milk from d 77 to 83 of age; total milk intake = 383 L, total milk solids intake = 65.1 kg), (3) HPM-12% TS (5 L/d of milk from d 4 to 13, 6 L/d of milk from d 14 to 23, 8 L/d of milk from d 24 to 69, and 5 L/d of milk from d 70 to 76 followed by feeding 3 L/d of milk from d 77 to 83 of age; total milk intake = 531 L, total milk solids intake = 63.7 kg); and 4) HPM-17% TS (5 L/d of milk from d 4 to 13, 6 L/d of milk from d 14 to 23, 8 L/d of milk from d 24 to 69, and 5 L/d of milk from d 70 to 76 followed by feeding 3 L/d of milk from d 77 to 83 of age; total milk intake = 531 L, total milk solids intake = 90.3 kg)

Calves were blocked by sex and randomly allocated to 1 of 4 dietary treatments (*n* = 12 calves, 6 male and 6 female calves per treatment; Fig. 1) in a 2 × 2 factorial arrangement with the factors of pre-weaning total PM intake (low vs. high) and milk TS content (12% vs. 17%). The 4 treatments were (1) low PM (LPM) intake with 12% TS (5 L/d milk from d 4 to 76 and 3 L/d of milk from d 77 to 83 of age; total milk intake = 383 L, total milk solids intake = 45.9 kg), (2) LPM intake with 17% TS (5 L/d milk from d 4 to 76 and 3 L/d of milk from d 77 to 83 of age; total milk intake = 383 L, total milk solids intake = 65.1 kg), (3) high plane of milk (HPM) intake with 12% TS (5 L/d of milk from d 4 to 13, 6 L/d of milk from d 14 to 23, 8 L/d of milk from d 24 to 69, and 5 L/d of milk from d 70 to 76 followed by feeding 3 L/d of milk from d 77 to 83 of age; total milk intake = 531 L, total milk solids intake = 63.7 kg); and (4) HPM intake with 17% TS (5 L/d of milk from d 4 to 13, 6 L/d of milk from d 14 to 23, 8 L/d of milk from d 24 to 69, and 5 L/d of milk from d 70 to 77 followed by feeding 3 L/d of milk from d 77 to 83 of age; total milk intake = 531 L, total milk solids intake = 90.3 kg).

Milk powder consisting of whey protein, dry skim milk and vegetable fat (96% DM, 25% CP, 25% ether extract, 9% ash, and 0.1% crude fiber; Soha Agreen Tech. Co., Tehran, Iran) was added to whole milk to increase milk TS concentration to 17%. Calves were weaned on d 83, and the study was terminated on d 103 of age.

The PMR diet was formulated for calves in the 60–70 kg BW range to achieve target ADG of 0.750 kg/d (Table 1) according to the Cornell Net Carbohydrate and Protein System, version 5.1 (CNCPS). A hammer mill with a 2-mm screen size (model 5543 GEN, Isfahan Dasht, Isfahan, Iran) was used to grind grains (corn and barley) in the starter feed. Calves received a blend of starter feed plus 8% chopped second-cut alfalfa hay as PMR throughout the study. Fresh water and PMR were offered for ad libitum intake to calves throughout the experimental period.

**Table 1:** Ingredients and chemical compositions of the experimental PMR

Item	% of DM basis
*Ingredients*	
Alfalfa hay	8.00
Corn grain, ground (flaked)	43.60
Barley grain, ground	5.43
Soybean meal (45% CP)	30.01
Fish meal	3.22
Corn hulls	4.60
Vitamin and mineral mix^2^	0.19
Sodium bicarbonate	1.84
Calcium carbonate	1.44
Dicalcium phosphate	0.28
Bentonite	0.46
MgO	0.44
Salt	0.49
*Chemical composition*	
ME,^3^ Mcal/kg	2.82
NEg,^3^Mcal/kg	1.24
DM	90.0
OM	91.1
CP	22.4
Ether extract	3.90
NDF	16.7
NFC^4^	49.2
Starch	38.4
Ca	1.11
P	0.61
Mg	0.47

^2^Contained per kilogram of supplement: 3,000,000 IU of vitamin A, 700,000 IU of vitamin D, 20,000 IU of vitamin E, 16,000 mg/kg of Mn, 20,000 mg/kg of Zn, 2,000 mg/kg of Fe, 90 mg/kg of Se, 150 mg/kg of Co, 8,000 mg/kg of Cu and 200 mg/kg of I

^3^Calculated according to NRC [73]

^4^Nonfiber carbohydrate was calculated as *DM* − (NDF + CP + ether extract + ash) [73]

### Data collection and sampling

The offered PMR and orts were measured daily to determine partial mixed ration (PMR) intake. Diets were sampled monthly and kept frozen (− 20 °C) for subsequent analyses. Dry matter was determined after oven-drying samples for 48 h at 65 °C, which then were ground through a 1-mm screen using a Wiley mill (Arthur Thomas Co., Philadelphia, PA). The ground samples were analyzed for N using Kjeldahl (method 988.05; AOAC [28]), ether extract (method 920.39; AOAC [28]), and ash (method 942.05; AOAC [28]). The neutral detergent fiber (NDF) and acid detergent fiber (ADF) contents were determined with the methods described by Van Soest et al. [29] using heat stable α-amylase.

Milk was sampled weekly from both the morning and evening meals, preserved with potassium dichromate, stored at 4 °C, and then analyzed for concentrations of fat, CP, lactose, and TS content by Milkoscan (Foss Electric, Hillerød, Denmark; AOAC International [30]). Calves were weighed at birth, 3 d after birth, and then every 10 d until the end of the experiment before the morning milk feeding. Total mixed ration intake, total DMI (milk plus PMR), average daily gain (ADG), and feed efficiency (kg BW gain per kg of total DMI) were calculated every 10 d throughout the study period (d 4–103). The morphometric measurements including body height, heart girth, body barrel, withers height, hip height, and hip width were measured as described previously [31] at the beginning, weaning, and end of study. Observational fecal score data were recorded daily before the morning milk feeding, on a scale of 0–3 according to the School of Veterinary Medicine calf health scoring chart, University of Wisconsin-Madison [32]. Fecal scores were established as 0 = normal, 1 = semi-formed and/or pasty, 2 = loose but stays on top of bedding, and 3 = watery and/or sifts through the bedding. Rectal temperature was measured every 10 d during the study using a thermometer (Qingdao Dacon Trading Co. Ltd., Shandong, China). Calves with fecal score ≥ 2 were considered to have diarrhea. All sick calves were diagnosed according to the standard operating procedures at the FKA Agriculture and Animal Husbandry Facility (Isfahan, Iran) and were treated with standard procedures prescribed by the veterinarian.

Calves with diarrhea under 2 weeks of age were treated with ScourSTOP (25 g per calf in milk for 1 d; Livestock Drugs Production Co., Garmsar, Iran) and water-based oral rehydration salt solution (2.5 L per calf; Sepid Dehdasht Co., Tehran, Iran). Older calves also received Enrofloxacin (Enrocin 5%; 3 mL per calf; Razak Laboratories Co., Karaj, Iran) and Flunixin meglumine (Flunixin 5%; 5 mL per calf; Razak Laboratories Co., Karaj, Iran). Also calves with pneumonia were treated with Florfenicol (10 mL per calf; Razak Laboratories Co., Karaj, Iran) and Flunixin meglumine (Flunixin 5%; 5 mL per calf; Razak Laboratories Co., Karaj, Iran) two times every second day. In case of no response, calves were treated two more days with Oxytetracycline (Oxivet 5%; 10 mL per calf; Razak Laboratories Co., Karaj, Iran) and Tylosin (Tyloject 20%; 10 mL per calf; Razak Laboratories Co., Karaj, Iran) or Ceftionel (Ceftiofur 5%; 6 mL per calf; Daanapharma Co., Tabriz, Iran).

Weather data including maximum and mean ambient temperatures and relative humidity were obtained from a meteorological station adjacent to the farm (about 1,000 m from the farm; Isfahan, Iran). Temperature-humidity index (THI) was calculated using the formula reported by Vitali et al. [33]: THI = 0.8 × maximum T + (minimum RH/100) × (maximum T − 14.4) + 46.4, where T is air temperature (°C) and RH denotes the relative humidity (%).

Ruminal fluid was obtained 4 h after the morning feeding with a stomach tube fitted to a vacuum pump on d 48 and d 91 of study; the first 100 mL was discarded to avoid saliva contamination as described by Shen et al. [34]. A sample of the fresh rumen liquid was used for pH measurement (HI 8318, Hanna Instruments, Cluj-Napoca, Romania). Then, 4 mL of the rumen fluid was acidified with 1 mL of 25% metaphosphoric acid and was stored (− 20 °C) until analysis for VFA by GC as described by Hashemzadeh-Cigari et al. [35]. For ammonia-N determination, a 2-mL subsample of filtered fluid was acidified with 2 mL of 0.2 mol/L HCl and frozen for subsequent analysis as described by Broderick and Kang [36].

Behavior was monitored visually by trained persons, unaware of treatments. Direct observations of all calves were made at pre-weaning (d 69 and 70) and post- weaning (d 93 and 94) under daylight conditions. Calves were observed for 12 h immediately following the morning feeding. Therefore, total observation time per animal was 24 h for the pre- and post-weaning monitoring periods. Four individuals were trained according to Kargar et al. [37] for monitoring behavior of calves, such that two persons observed the calves and the other two persons rested simultaneously for 2 h periods. Calves were observed every 5 min and each activity was assumed to persist for the entire 5 min interval between observations. The observers recorded the occurrence of the following behaviors: lying (no chewing activity), standing (no chewing activity), eating feed, ruminating (either lying or standing), and non-nutritive oral behaviors (NNOB; when the animal licked any surface or tongue rolled). A bout was defined as at least one observation of a specific activity occurring after a different activity [37]. The number of bouts during each 12 h period was defined as bout frequency and the bout duration (min/bout) was calculated as the averaged time from the beginning of each activity occurrence until another activity occurrence for each calf [37].

### Statistical analysis

Data were checked for normality using the UNIVARIATE procedure (SAS 9.2, SAS Institute Inc., Cary, NC). The data that were not normally distributed, including feed efficiency, body length, hearth girth, acetate concentration, eating time and NNOB, were transformed logarithmically. Data for PMR, total DM and ME intakes recorded daily were first averaged over every 10 day, and then PMR, total DM and ME intakes, BW, ADG, feed efficiency, skeletal growth, ruminal fermentation parameters, behavioral measurements and rectal temperature data were analyzed as repeated measures, with period (10 d periods or sampling times) as the repeated variable using the model:

$$ Yijkl=\mu + Ai+ Pj+ TSk+ Tl+\left(P\times T\right) jl+\left( TS\times T\right) kl+\left(P\times TS\right) jk+\left(P\times TS\times T\right) jk l+\beta \left( Xi-X\right)+\varepsilon ijkl $$

The disease incidence and medication days were analyzed using the model:

$$ Y\  ijl=\mu + Pi+ TSj+\left(P\times TS\right) ij+\varepsilon ijl $$

where *Yijkl* is the dependent variable; *μ* is the overall mean; *Ai* is the random effect of calf; *Pj* is the fixed effect of PM; *TSk* is the fixed effect of milk total solid; *Tl* is the fixed effect of period; (*P* × *T*) *jl* is the interaction between PM and period; *(TS × T) kl* is the interaction between milk TS and period; *(P × TS) jk* is the interaction between PM and milk TS; *(P × TS × T) jkl* is the tripartite effect of PM, milk TS, and period; *β(Xi − X)* is the covariate variable (for BW and skeletal growth, the initial values were considered as covariates); and *εijkl* is the random residual error. The effect of dietary treatment on the categorical responses related to fecal score was tested using the GLIMMIX procedure of SAS version 9.4. A covariance structure (unstructured, compound symmetry heterogeneous, autoregressive order 1, or ante-dependence order 1) was chosen based on the lowest Akaike information criterion and Bayesian information criteria indexes. Significance was declared at *P* < 0.05 and trends were considered when 0.05 < *P* < 0.10.

## Results

**Fig. 2:**
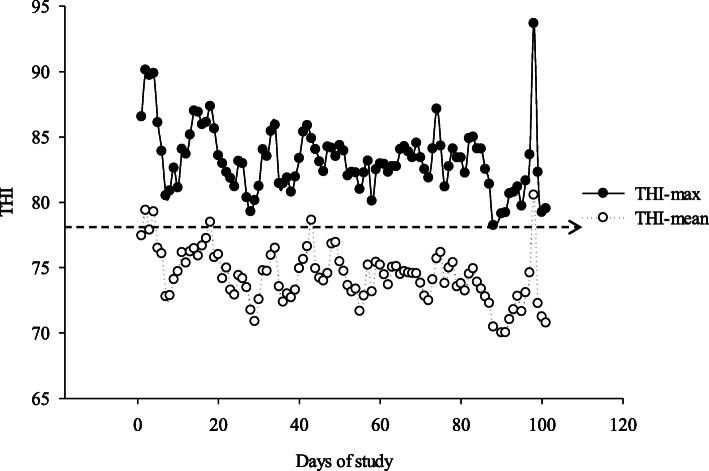
Temporal patterns of mean and maximum (max) temperature-humidity index (THI) over the experimental period, with average values of 74.42 ± 2.02 and 83.3 ± 2.51, respectively. The dashed line (THI ≥ 78) represents the threshold for moderate to severe thermal stress [74]

**Fig. 3:**
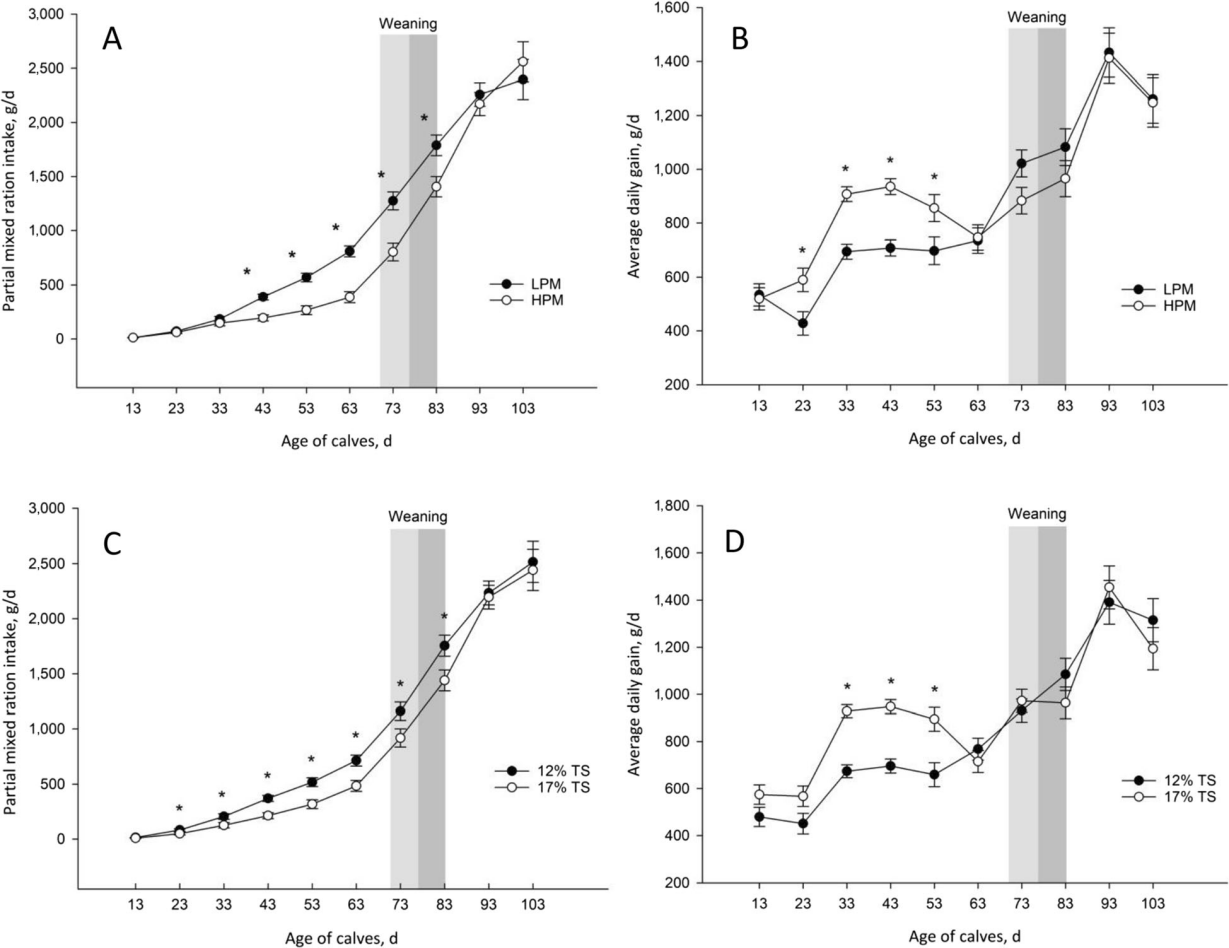
Effects of two-way interactions between different PM (LPM vs. HPM) × age and milk TS (12% vs. 17%) × age on PMR intake (**A** and **C**) and ADG (**B** and **D**) during the study. Light gray period: gradual weaning (5L/d from d 70 to 76 of age); Dark gray period: gradual weaning (3L/d from d 77 to 83 of age). Weaning period for HPM included light and dark gray and for LPM included only dark gray. Asterisks indicate significant differences (*P* < 0.05) at corresponding period. Error bars denoted SEM

**Fig. 4:**
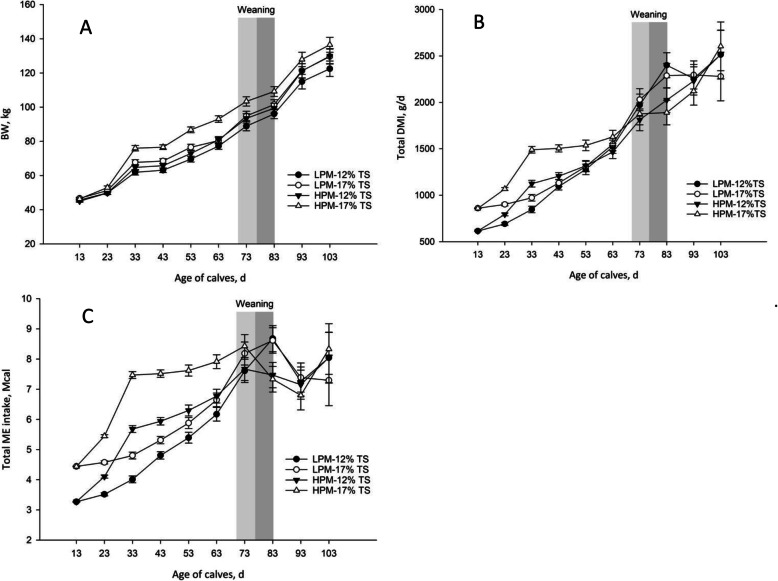
Effects of three-way interactions among different PM (LPM vs. HPM) milk TS (12% vs. 17%) × age on BW (**A**), total DMI (**B**) and total ME intake (**C**) during the study. BW was greater (*P* = 0.03) for calves fed HPM-17% TS versus others from d 14 to 103 of age. TDMI (*P* = 0.003) and ME (*P* = 0.0001) were differed among all treatments during d 14 to 43 of age. Total DMI and ME intake were greater for LPM-12% and LPM-17% versus HPM-12% and HPM-17% during d 74 to 83 of age. Light gray period: gradual weaning (5L/d from d 70 to 76 of age); Dark gray period: gradual weaning (3L/d from d 77 to 83 of age). Weaning period for HPM included light and dark gray and for LPM included only dark gray. Error bars denoted SEM

The maximum and mean THI values were relatively consistent throughout the experiment, with average values of 83.3 ± 2.51 and 74.42 ± 2.02, respectively (Fig. 2). Data on milk TS intake and performance measurements are summarized in Table 2. As designed, milk TS and milk ME intakes were lowest for calves receiving LPM-12% TS, intermediate for those receiving LPM-17% TS and HPM-12% TS, and greatest for those receiving HPM-17% TS. For PMR and PMR ME intakes, ADG and feed efficiency, we detected a PM intake × age interaction (*P* < 0.001). The PMR intake (Fig. 3) and PMR ME intake were greater in LPM-fed calves than HPM-fed calves from 44 to 83 d of age (*P* < 0.0001). There was a three way PM × milk TS × age interaction for total DMI *(P* = 0.003; Fig. 4A) as follows: total DMI was greater for HPM-17% and LPM-17% versus LPM-12% and HPM-12% during d 4–13 of age, differed among all 4 treatments where LPM-12% < HPM-12% < LPM-17% < HPM-17% during d 14–23 of age and LPM-12% < LPM-17% < HPM-12% < HPM-17% during d 24–33 of age, greater for HPM-17% versus other treatments during d 34–53 of age, greater for LPM-12% and LPM-17% versus HPM-12% and HPM-17% during d 74–83 of age and no effects of treatments was observed from d 84–103 of age. A similar three way interaction was also detected for total ME intake (*P* = 0.0001; Fig. 4C). As shown in Fig. 3B, calves receiving HPM had higher ADG from d 24–53 of age (*P* < 0.05), but from d 64–73, these calves had lower ADG than those fed LPM (*P* = 0.06). A three-way interaction was identified among PM intake, milk TS and age for BW (*P* = 0.03; Fig. 4B) whereby during d 14–103 of age, BW was greater in calves fed HPM-17% versus other treatments, and no differences observed between HPM-12% and HPM-17 during d 14–103 of age. Although feed efficiency was not affected by PM feeding, the PM feeding × age interaction (*P* = 0002) showed that feed efficiency was greater in calves fed HPM compared to those fed LPM from d 24–43 of age. Moreover, the interaction between milk TS content and age was significant for PMR and PMR ME intakes, ADG and feed efficiency. The PMR intake (Fig. 3C) and PMR ME intake were greater for calves offered milk with 12% versus 17% TS (*P* < 0.05) during 24–83 d of age. Conversely, ADG (Fig. 3D) and feed efficiency during d 24–53 were greater in calves that received milk with 17% TS compared to those that received milk containing 12% TS (*P* < 0.05).

**Table 2:** Effects of milk plane and milk total solid content on overall performance parameters of dairy calves during the study

Items	LPM^2^		HPM^2^		SEM^1^	*P* values						
	12	17	12	17		PM	TS	PM × TS	Age (A)	PM × A	TS × A	PM × TS × A
Milk TS intake, kg	45.9	65.1	63.7	90.3	–	–	–	–	–	–	–	–
Milk ME intake^3^, Mcal	3.12^d^	4.28^c^	4.34^b^	5.95^a^	0.008	0.0001	0.0001	0.0001	–	–	–	–
PMR intake, g/d	1,051.9	899.5	862.3	739.9	67.37	0.01	0.05	0.82	< 0.0001	< 0.0001	0.004	0.33
Total DMI^4^, g/d	1,519.2	1,561.8	1,510.6	1,657.9	67.06	0.52	0.16	0.44	< 0.0001	< 0.0001	0.05	0.003
Total ME intake, Mcal	5.9	6.3	6.2	7.1	0.21	0.01	0.003	0.29	< 0.0001	< 0.0001	0.01	0.0001
BW^5^, kg	78.9	83.8	82.3	90.9	1.90	0.01	0.001	0.33	< 0.0001	< 0.0001	< 0.0001	0.03
PMR ME intake, Mcal	3.4	2.9	2.8	2.4	0.22	0.01	0.05	0.82	< 0.0001	< 0.0001	0.004	0.33
ADG6, g/d	830.4	888.8	859.3	953.5	37.32	0.22	0.05	0.64	< 0.0001	< 0.0001	< 0.0001	0.24
Feed efficiency^7^	0.59	0.59	0.58	0.60	0.01	0.79	0.39	0.67	< 0.0001	0.0002	0.01	0.48

^1^Standard error of the means

^2^(1) calves fed low plane of milk (LPM) intake with 12% TS (LPM-12%TS; total milk intake = 383 L; TS intake = 45.9 kg), (2) calves fed LPM intake with 17% TS (LPM-17%TS; total milk intake = 383 L; TS intake = 65.1 kg), (3) calves fed high plane of milk (HPM) intake with 12% TS (HPM-12%TS; total milk intake = 531 L; TS intake = 63.7 kg); and (4) calves fed HPM intake with 17% TS (HPM-17%; total milk intake = 531 L; TS intake = 90.3 kg)

^3^*ME* Metabolizable energy

^4^*DMI* Dray matter intake

^5^*BW* Body weight

^6^*ADG* Average daily gain

^7^Feed efficiency was calculated from dividing ADG (g/d) by daily DMI (g/d)

^a^^−^^c^Means within a row with different superscripts differ significantly (*P* < 0.05)

Overall means of skeletal growth parameters are presented in Table 3. Calves that received HPM had greater body length (62.8 vs. 61.1 cm; *P* = 0.04), hip height (103.3 vs. 102.0 cm; *P* = 0.04) and hip width (19.8 vs. 19.1 cm; *P* = 0.01) and tended to have greater body barrel (131.4 vs. 128.4 cm, *P* = 0.07) and withers height (100.0 vs. 98.9 cm; *P* = 0.09) compared to LPM-fed calves. Also, overall means of body length (63.7 vs. 60.3 cm; *P* < 0.01), heart girth (114.1 vs. 109.4 cm; *P* = 0.01), body barrel (131.4 vs. 128.1 cm; *P* = 0.03), withers height (100.4 vs. 98.4 cm; *P* < 0.01), hip height (103.8 vs. 101.5; *P* < 0.01), and hip width (19.8 vs. 19.1 cm; *P* = 0.01) were greater in calves fed milk with 17% TS than in those fed milk with 12% TS (*P* < 0.05). As expected, skeletal growth parameters increased as calves aged. However, no interactions were observed for the PM intake × milk TS, PM intake × age, milk TS × age (*P* > 0.10) or PM intake × milk TS × age with respect to skeletal growth measures.

**Table 3:** Effects of milk plane and milk total solid content on overall skeletal growth parameters of dairy calves during the study

Items	MPM^2^		HPM^2^		SEM^1^	*P* values						
	12	17	12	17		PM	TS	PM × TS	Age (A)	PM × A	TS × A	PM × TS × A
Body length, cm	59.9	62.3	60.7	64.9	0.79	0.04	< 0.0001	0.25	< 0.0001	0.91	0.97	0.89
Heart girth, cm	110.1	113.5	109.3	114.7	1.58	0.89	0.01	0.50	< 0.0001	0.24	0.67	0.35
Body barrel, cm	127.2	129.7	128.9	133.7	1.64	0.07	0.03	0.49	< 0.0001	0.44	0.48	0.28
Withers height, cm	98.0	99.7	98.8	101.1	0.64	0.09	0.003	0.65	< 0.0001	0.74	0.74	0.68
Hip height, cm	101.1	102.9	101.9	104.6	0.62	0.04	0.0005	0.55	< 0.0001	0.99	0.65	0.99
Hip width, cm	18.7	19.6	19.6	20.1	0.28	0.01	0.01	0.47	< 0.0001	0.84	0.41	0.43

^1^Standard error of the means

^2^(1) calves fed low plane of milk (LPM) intake with 12% TS (LPM-12%TS; total milk intake = 383 L; TS intake = 45.9 kg), (2) calves fed LPM intake with 17% TS (LPM-17%TS; total milk intake = 383 L; TS intake = 65.1 kg), (3) calves fed high plane of milk (HPM) intake with 12% TS (HPM-12%TS; total milk intake = 531 L; TS intake = 63.7 kg); and (4) calves fed HPM intake with 17% TS (HPM-17%; total milk intake = 531 L; TS intake = 90.3 kg)

Ruminal fermentation measurements are shown in Table 4. There was an interaction between PM intake and age of calves for NH_3_–N (*P* = 0.01), such that calves receiving HPM had higher rumen NH_3_–N concentration than those receiving LPM on d 48 and 91, but this response was more pronounced on d 48 versus 91 (+ 122% vs. + 43%). Interaction between PM feeding and milk TS content tended to be significant for ruminal pH (*P* = 0.08) and butyrate (*P* = 0.10) where rumen pH was greater in calves receiving milk with 17% TS versus 12% TS only in LPM-fed calves, but butyrate concentration was greater in calves receiving milk with 17% TS versus 12% TS only in HPM-fed calves. In addition, we detected interactions between PM intake and age of calves for isobutyrate (*P* = 0.002) and isovalerate (*P* = 0.002) concentrations, which implied that calves fed HPM had greater isobutyrate and isovalerate concentrations than calves fed LPM on d 48, but this response was not observed at d 91 of age. Regardless of dietary treatments, rumen total VFA, propionate and valerate concentrations increased, while rumen pH, acetate, butyrate, isobutyrate, isovalerate, NH_3_ and acetate to propionate ratio decreased as calves aged (*P* < 0.05). No three-way interactions were identified between PM intake, milk TS and age (*P* > 0.10) for any ruminal parameters.

**Table 4:** Effects of milk plane intake and total solids of milk on overall ruminal variables of dairy calves

Items	LPM^2^		HPM^2^		SEM^1^	*P* values						
	12	17	12	17		Milk	TS	PM × TS	Age (A)	PM × A	TS × A	PM × TS × A
NH_3_-N, mmol/L	6.42	8.97	15.20	13.64	1.31	< 0.0001	0.71	0.14	< 0.0001	0.01	0.81	0.62
Ruminal pH	5.32	5.52	5.59	5.48	0.08	0.19	0.57	0.08	< 0.0001	0.95	0.33	0.63
Total VFA, mmol/L	122.64	122.71	108.87	116.11	10.12	0.34	0.73	0.73	< 0.0001	0.13	0.17	0.44
Acetate, mmol/L	50.01	51.54	52.91	52.96	1.89	0.28	0.69	0.71	< 0.0001	0.43	0.09	0.90
Propionate, mmol/L	40.49	38.70	37.59	36.33	1.77	0.16	0.41	0.88	< 0.0001	0.42	0.19	0.53
Butyrate, mmol/L	6.61	6.11	5.69	7.14	0.55	0.92	0.42	0.10	< 0.0001	0.51	0.99	0.39
Isobutyrate, mmol/L	0.14^c^	0.36^b^	0.58^a^	0.45^ab^	0.08	0.003	0.62	0.04	< 0.0001	0.002	0.30	0.46
Valerate, mmol/L	2.18	2.67	2.17	2.52	0.31	0.80	0.22	0.83	0.05	0.54	0.36	0.33
Isovalerate, mmol/L	0.34	0.58	0.96	0.74	0.13	0.01	0.94	0.11	< 0.0001	0.002	0.27	0.82
Branched chain VFA, mmol/L	2.69	3.62	3.73	3.72	0.42	0.20	0.29	0.29	0.29	0.19	0.23	0.34

^1^Standard error of the means

^2^(1) calves fed low plane of milk (LPM) intake with 12% TS (LPM-12%TS; total milk intake = 383 L; TS intake = 45.9 kg), (2) calves fed LPM intake with 17% TS (LPM-17%TS; total milk intake = 383 L; TS intake = 65.1 kg), (3) calves fed high plane of milk (HPM) intake with 12% TS (HPM-12%TS; total milk intake = 531 L; TS intake = 63.7 kg); and (4) calves fed HPM intake with 17% TS (HPM-17%; total milk intake = 531 L; TS intake = 90.3 kg)

^a–c^Means within a row with different superscripts differ significantly (*P* < 0.05)

Table 5 presents the times apportioned to eating, ruminating, standing, lying, drinking activities, and NNOB under daylight condition. There was an interaction between PM intake and age on eating time (*P* = 0.01) and lying time (*P* = 0.04). This interaction indicated that eating time was greater (55.4 vs. 45.6 min; *P* = 0.03) for calves receiving LPM versus HPM on d 69 to 70, but contrasting results (82.7 vs. 102.2 min; *P* = 0.07) were observed on d 93–94 of age. Also, times apportioned to lying tended to be greater (374.2 vs. 352.4 min; *P* = 0.07) in calves receiving HPM compared to those receiving LPM only at d 69–70 of age. The time devoted to drinking was greater for calves receiving HPM than those receiving LPM (17.74 vs. 12.97 min; *P* = 0.04). Also, a tendency for interaction was observed between milk TS content and age for drinking time (*P* = 0.06), such that calves fed milk with 17% TS spent more time drinking (22.82 vs. 11.83 min; *P* = 0.01) than those fed milk with 12% TS only at d 93–94 of age. Finally, times spent ruminating, standing and in NNOB were not affected by PM intake, milk TS content, or their interaction during the study.

**Table 5:** Effects of milk plane intake and total solid of milk on overall behavior of dairy calves under daylight condition

Items	LPM^2^		HPM^2^		SEM^1^	*P*-value						
	12	17	12	17		PM	TS	PM × TS	Age (A)	PM × A	TS × A	PM × TS × A
*Time apportioned, min/12 h*												
Eating	71.52	66.75	74.98	72.88	5.62	0.39	0.54	0.81	< 0.0001	0.01	0.53	0.38
Ruminating	116.51	128.42	115.74	115.54	8.44	0.42	0.49	0.47	0.002	0.47	0.18	0.21
Standing	131.95	121.00	136.77	134.59	8.27	0.27	0.43	0.60	0.71	0.49	0.87	0.33
Lying	333.28	337.05	329.89	330.16	12.26	0.68	0.87	0.89	< 0.0001	0.04	0.21	0.51
NNOB^3^	56.02	51.55	49.33	44.62	6.95	0.33	0.51	0.99	0.34	0.48	0.73	0.56
Drinking	10.72	15.23	13.29	22.20	2.31	0.04	0.005	0.34	0.09	0.12	0.06	0.52
*Bout frequency, bouts/12 h*												
Eating	8.01	8.23	9.41	7.88	0.7	0.45	0.35	0.21	0.0002	0.13	0.51	0.66
Ruminating	11.63	12.93	11.47	13.52	0.69	0.75	0.01	0.59	0.25	0.13	0.59	0.36
Standing	16.57	15.68	15.47	16.04	0.50	0.46	0.74	0.15	0.0004	0.05	0.03	0.32
Lying	16.23	16.08	15.70	16.30	0.53	0.77	0.67	0.49	0.003	0.15	0.12	0.60
*Bout duration, min/bout*												
Eating	9.24	9.05	8.39	8.85	0.63	0.41	0.83	0.61	< 0.0001	0.43	0.21	0.72
Ruminating	10.40	10.06	10.49	8.52	0.58	0.22	0.05	0.17	< 0.0001	0.60	0.86	0.37
Standing	8.09	7.85	9.05	8.88	0.7	0.16	0.77	0.96	0.16	0.14	0.41	0.60
Lying	21.16	21.39	21.76	20.63	1.13	0.94	0.69	0.55	0.17	0.74	0.94	0.32

^1^Standard error of the means

^2^(1) calves fed low plane of milk (LPM) intake with 12% TS (LPM-12%TS; total milk intake = 383 L; TS intake = 45.9 kg), (2) calves fed LPMintake with 17% TS (LPM-17%TS; total milk intake = 383 L; TS intake = 65.1 kg), (3) calves fed high plane of milk (HPM) intake with 12% TS (HPM-12%TS; total milk intake = 531 L; TS intake = 63.7 kg); and (4) calves fed HPM intake with 17% TS (HPM-17%; total milk intake = 531 L; TS intake = 90.3 kg)

^3^NNOB Non-nutritive oral behaviors refer to licking any surface, tongue rolling, etc.

Data on bout frequency and bout duration for eating, ruminating, standing, and lying are presented in Table 5. Calves receiving 17% TS had more ruminating bouts than those that received 12% TS (13.22 vs. 11.55; *P* = 0.01). Interactions between PM intake and age and milk TS content and age were significant for standing bout frequency, whereby standing frequency was higher in calves fed LPM vs. HPM (15.70 vs. 14.34; *P* = 0.06) and in calves fed milk with 12% TS versus 17% TS (15.65 vs. 14.39; *P* = 0.05) only at d 93–4 of age. The PM intake, milk TS content and their interaction did not affect behavioral bout durations, except for rumination bout duration, which was shorter in calves offered milk with 17% TS (9.28 vs. 10.44 min; *P* = 0.05) compared to those fed milk with 12% TS. No three-way interactions were identified between PM × milk TS × age (*P* > 0.10) for any behavioral parameters.

Table 6 presents the fecal score, rectal temperature, occurrence of diarrhea (fecal score ≥ 2) and pneumonia and medical days during the study. A tendency for interaction (*P* = 0.06) between the effect of milk plane and milk TS was detected for fecal score, indicating that calves receiving milk with 17% TS had greater fecal score compared to those fed milk with 12% TS in HPM, but not LPM diets. Moreover, interaction between milk TS content and age (*P* = 0.04) for fecal score revealed that calves receiving milk with 17% TS had higher fecal score from d 4 to 18 of age compared to those fed milk with 12% TS, but there was no difference thereafter. Although rectal temperature was not affected by PM intake, milk TS content or their interaction, an interaction was detected between milk TS content and age (*P* = 0.03) such that rectal temperature was higher for calves fed milk with 17% TS than those fed milk with 12% TS at d 14 of age, while contrasting response was observed at d 43 of age.

**Table 6:** Effects of milk plane intake and total solid of milk on overall health parameters during pre-weaning period

Items	LPM^2^		HPM^2^		SEM^1^	*P* values						
	12	17	12	17		PM	TS	PM × TS	Age (A)	PM × A	TS × A	PM × TS × A
Fecal score^3^	0.12	0.13	0.08	0.17	0.02	0.92	0.01	0.06	< 0.0001	0.44	0.04	0.91
Rectal temperature	38.96	38.88	38.96	39.99	0.047	0.25	0.61	0.35	0.01	0.66	0.03	0.22
Diarrhea occurrence, d	1.96	2.17	1.33	2.92	0.33	0.85	0.01	0.04	–	–	–	–
Pneumonia occurrence, d	0.67	0.37	0.50	0.42	0.16	0.71	0.26	0.53	–	–	–	–
*Medical days*												
Electrolytes	2.75	3.17	2.00	4.67	0.35	0.56	0.02	0.08	–	–	–	–
Diarrhea medicine	4.33	5.50	3.17	7.50	0.54	0.67	0.01	0.10	–	–	–	–
Pneumonia medicine	5.75	6.25	4.25	6.58	0.50	0.53	0.13	0.33	–	–	–	–
Other medicine	2.50	1.92	1.08	1.58	0.24	0.07	0.93	0.26	–	–	–	–

^1^Standard error of the means

^2^(1) calves fed low plane of milk (LPM) intake with 12% TS (LPM-12%TS; total milk intake = 383 L; TS intake = 45.9 kg), (2) calves fed LPMintake with 17% TS (LPM-17%TS; total milk intake = 383 L; TS intake = 65.1 kg), (3) calves fed high plane of milk (HPM) intake with 12% TS (HPM-12%TS; total milk intake = 531 L; TS intake = 63.7 kg); and (4) calves fed HPM intake with 17% TS (HPM-17%; total milk intake = 531 L; TS intake = 90.3 kg)

^3^Fecal scores were established as a scale 0–3 (0 = normal, 1 = semi-formed and/or pasty, 2 = loose but stays on top of bedding, and 3 = watery and/or sifts through the bedding) using the Wisconcin-Madison’s Calf Health Scoring chart [32]

^a–c^Means within a row with different superscripts differ significantly (*P* < 0.05)

An interaction was observed between PM intake and milk TS for diarrhea occurrence, with the greatest (*P* = 0.04) incidence recorded in calves that received HPM-17% compared to other treatments. Calves fed milk with 17% TS received more medication for diarrhea (*P* = 0.01) and electrolytes (*P* = 0.02) than those fed milk with 12% TS, but these responses were more pronounced when calves were offered HPM (interaction, *P* = 0.09). Calves fed HPM diets tended to have lower (*P* = 0.07) number of days treated with other veterinary medications than those receiving LPM.

## Discussion

Summer conditions may negatively affect welfare, liquid and solid feed intakes, health and thermoregulatory responses, and growth performance of calves, resulting in impaired short- and long-term survival and productivity [8]. Calves receive most of their nutrients from milk or MR during the pre-weaning period and heat stress severely impairs starter intake (− 52%; [38]). Therefore, increasing the allowance of liquid feed or total solids in liquid feed to calves, particularly in the first weeks of life, would be an effective strategy to increase available nutrients for growth [22].

Information regarding accelerated milk feeding programs (more than 0.80 kg total solid per day) in late-weaned calves during summer conditions is limited. Therefore, in the present study, we investigated the effects of offering greater amounts of milk TS to environmentally heat-loaded dairy calves by two methods, either (1) higher amounts of normal TS milk, or (2) moderate amounts of higher TS milk.

Based on our findings, feeding higher amounts of milk to calves had significant effects on performance responses during the pre-weaning period. The PMR intake was similar between the two PM until d 43 of age but was lower in calves receiving higher PM from d 44 to weaning at d 83 of age compared to those received LPM. In a meta-analysis study, feeding higher amounts of MR to early-weaned calves (at d 56 of age) resulted in lower feed intake during the pre-weaning period and similar starter intake from d 56 to 84 [14]. In addition, Bach et al. [39] reported that feeding large amounts of MR to late-weaned calves resulted in lower starter intake until d 52 and similar starter intake from d 53 to weaning at d 72 of age. These results might be indicative that during summer conditions in the present study, the negative effects of HPM feeding on PMR intake were observed in older age (from d 44 of age) and continued until weaning at d 83 of age compared to LPM. Recently, Orellana Rivas et al. [3], based on higher bloat incidence in calves fed 0.87 DM of MR, suggested that feeding 0.87 kg DM of MR divided into 2 feedings daily to summer raised-calves would likely delay abomasal emptying rate due to large meal size and heat stress, which may compromise health and intake. Moreover, lower PMR intake in calves fed HPM could be related to the ingestion of highly nutrient-dense milk, which meets most of the calf's nutrient requirements, leaving little appetite for starter intake [40]. As provisioned, intake of total milk solids for different feeding treatments during pre-weaning period were in the following order: 17%-HPM > 17%-LPM = 12%-HPM > 12%-LPM with means of 90.3 kg, 65.1 kg, 63.7 kg and 45.9 kg, respectively, which was similar to the order that was observed for pre-weaning total ME intake and consequently BW of calves.

Calves fed HPM had higher feed efficiency from d 24 to 43 of ages and ADG from d 24 to 53 compared to calves fed LPM, whereas PMR intake was similar and TME intake was in the following order: 17%-HPM > 12%-HPM > 17%-LPM > 12%-LPM during this period. At the same time, regarding lower PMR intake in calves fed milk with 17% TS, they had greater feed efficiency and ADG than calves fed milk with 12% TS during d 24–53 of ages. These results are likely to be related to the considerably higher digestibility of milk in comparison to starter feed and was in line with previous reports [17, 41–44]. Another notable result during this period was that calves fed 17%-HPM and 12%-HPM received higher TME intake than calves fed 17%-LPM and 12%-LPM, which indicates that feed efficiency was more closely related to PM than TS of milk [43]. These results are inconsistent with the results of Azevedo et al. [25] who reported that higher amounts of TS in milk due to replacement of nutrients from a dairy source with a non-dairy source decreases feed efficiency.

During the weaning period (from 71 to 83 d of age), calves fed HPM-17% had the highest BW whereas TDMI and TME were in the following order: HPM-17% = HPM-12% < LPM-12% and HPM-17% < LPM-17%. Regardless of treatment, weaning caused a significant increase in PMR intake in all the calves, which indicates that calves were able to utilize nutrients from PMR at this age in line with previous reports [45, 46]. These results are in agreement with studies that indicate beneficial effects of delayed weaning in calves fed HPM on intake and growth during weaning and pre-weaning periods [16, 19]. However, Dennis et al. [17] showed that growth performance did not improve in calves fed high rates of MR and weaned 1 week later, which differs from the findings presented here. In the current study, final BW was highest in calves fed HPM-17% TS, which were greater than calves fed LPM-12% TS.

Intakes of PMR and ME were similar between the 2 PM treatments from d 83 to 103 of age. This means that despite limited PMR intake of HPM-fed calves during the first 2-month of age, decreasing milk provision during the weaning transition (d 69–83 of age) progressively increased PMR intake until weaning and thereafter, which allowed them to compensate for the poorer starter intake early in life. On the other hand, calves fed LPM experienced a slight slump of PMR intake during d 94–103 of age. Because ADG were similar between calves fed either PM feeding rates from d 83 to 103 of age, we speculate that feeding HPM in an extended gradual weaning protocol may help calves to adopt and successfully transition from this critical period [20, 47] especially when they are exposed to hot weather conditions. Our results showed that PMR intake was lower in calves fed milk with 17% TS concentration than those fed milk with 12% TS concentration during d 14–83 of age. In agreement with our results, Glosson et al. [43] reported that addition of milk balancer to whole milk decreased starter intake; however, others showed that increasing milk TS concentration with MR up to 18.2% did not affect starter intake during the pre-weaning period [25, 48]. Moreover, our results demonstrated that calves fed LPM-17% TS had greater PMR intake than calves fed HPM-12% TS (699.1 vs. 505.6 g/d; *P* = 0.06) from d 64 to 73 of age. This means that although milk TS intake was similar for these two groups, route of feeding may result in different responses in starter diet intakes of calves.

Similar to BW, body length, hip height and hip width were increased by the accelerated milk feeding program along with body barrel and withers height, which tended to be greater in calves fed HPM than in those fed LPM. In agreement with our results, other researchers reported that feeding HPM to early weaned-calves resulted in greater growth performance than those fed LPM during the pre-weaning period [18, 21, 49]. Also, our results suggest that greater intakes of ME and other nutrients derived from higher milk provision mostly shifted toward body skeletal growth rather than adipose tissues development, which is in line with previous reports [11, 50, 51]. Interestingly, skeletal growth measurements were greatly affected by milk TS content, so that calves that received milk with 17% TS had greater BW and skeletal growth parameters, including body length, heart girth, body barrel, withers height, hip height, and hip width compared to those fed milk with 12% TS concentration during the study. These results are in good agreement with other researchers, who reported that BW and some skeletal growth parameters were enhanced in response to increasing milk TS concentration [25, 48]. Results of the present study showed that enhanced growth measurements during the post-weaning period were mostly related to growth promotion during the pre-weaning period because post-weaning growth responses were similar between calves fed two milk TS concentrations.

In the present study, overall concentrations of acetate, propionate, butyrate, valerate and total VFA in ruminal fluid were similar during the study for calves fed HPM and LPM, indicative that rumen function was probably not different between them. However, isobutyrate and isovalerate were greater in calves that received HPM compared to those fed LPM at d 48 of age. In agreement with our results, Koch et al. [52] showed that MR feeding rate did not affect ruminal fermentation characteristics, except for isobutyrate and isovalerate concentration, which increased in calves fed MR for ad libitum intake compared to those fed a restricted amount of milk. Also, rumen NH_3_-N concentration was greater at both sampling days for calves offered HPM compared to those that were fed LPM, which is in accordance with previously published studies [53, 54]. Moreover, in the current study, there were strong correlations between ruminal NH_3_-N concentration and isobutyrate (r = 0.91; *P* < 0.0001) and isovalerate (r = 0.91; *P* < 0.0001) concentrations. Milk proteins, especially casein, contain a higher proportion of branched-chained amino acid than soybean meal as the main protein source in the starter diet [55]. It is possible that bucket-feeding higher amounts of milk with limited frequency increased milk leaking into the rumen to be fermented by the ruminal microbes, resulting in greater NH_3_-H and branched chain-VFA in the rumen. On the other hand, after weaning, lower ruminal NH_3_-N in LPM fed calves might be attributed to (1) the greater ruminal NH_3_-N absorption due to lower ruminal pH, or (2) more rumen fermentation, which incorporated more NH_3_-N to microbial protein biomass, resulting in lower NH_3_-N concentration in the rumen fluid.

Ruminal total VFA concentration increased, while rumen pH, acetate and isobutyrate decreased as calves aged, which could be partly attributed to higher PMR intake of calves over time. Greater consumption of starter diet by calves around weaning may foster the initiation of rumen fermentation and accelerate rumen development, which can confer benefits of gradual and late weaning to stimulate earlier solid feed intake and its importance for gastrointestinal tract development [18, 56]. The results in the present study are in agreement with Khan et al. [21], who showed weaning through step-down method caused greater consumption of solid feed in calves fed large amounts of milk and resulted in early initiation of ruminal fermentation and heavier forestomach compared with those fed milk conventionally. Moreover Meale et al. [57] showed that delayed weaning in calves fed a high-plane of pre-weaning nutrition facilitates a more gradual shift in rumen microbial diversity, which could explain the negative effects of early-weaning. These results may indicate that offering a greater volume of milk and milk TS concentration has minor effects on ruminal development when calves consume small amounts of PMR diet. However, during the early post-weaning period, intensive milk feeding programs may interfere with the onset of ruminal fermentation and functionality [21].

In the present study, ruminal fermentation parameters were not affected by milk TS concentration, except for butyrate concentration, which increased in calves fed HPM-17% TS treatment. In agreement with our results, Azevedo et al. [48] showed that ruminal pH, NH_3_–N concentration, VFA proportions, and total VFA concentration were not affected by incremental milk TS concentrations. These results suggest that enhancing nutrient provision to calves via increasing milk TS concentration did not adversely affect rumen fermentation characteristics, helping them to smoothly pass the weaning period.

Milk feeding programs can affect the feeding behavior of dairy calves [58]. At d 69–70 of age, calves that received the larger amount of milk apportioned more time to lying at the expense of starter eating time. In agreement with our results, Miller-Cushon et al. [59] reported that providing ad libitum amounts of milk resulted in less frequent, smaller starter feed meals than those fed restricted amounts of milk before weaning. Previously, it has been suggested that chemical stimuli, such as higher blood glucose and insulin, may exert hypophagic effects [21]. Therefore, higher glucose concentration in response to feeding a higher volume of milk may stimulate satiety signals, resulting in lower eating time. Moreover, calves fed large amounts of milk have previously been shown to spend less time standing and more time lying down than restricted-fed calves [60, 61]. In general, lying time in young calves is considered as an indicator of comfort. Further, calves expend less energy during lying time [62]. Therefore, feeding a greater amount of milk in the present study may enhance calves' comfort and energy status during summer conditions. At d 93–94 of age, calves previously fed the HPM apportioned more time for consuming PMR and drinking water than those fed the LPM, which could be related to their higher hunger level. Calves fed LPM versus HPM and 12% TS verus 17% TS had greater standing frequencies at d 93–94 of age. Previously, it has been reported that calves weaned abruptly displayed more standing bouts compared to those with continued access to the milk-feeding apparatus, indicating that these calves experienced more weaning distress [63]. Thus, more standing frequency in the calves fed LPM-12% TS might be related to some degrees of discomfort during the pre-weaning period. As discussed earlier, these calves had more extensive ruminal fermentation and experienced acidosis condition, as evidenced by lower ruminal pH, which could be related to the more frequent standing behavior. DeVries et al. [64] reported that acute bouts of acidosis challenge alters some behavioral patterns such as more standing frequency of lactating dairy cows. Our results demonstrated that despite the similar overall rumination time in calves fed milk with higher TS concentration, they had shorter overall ruminating bout duration but more overall ruminating frequency and greater time apportioned to drinking water only during d 93 to 94 of age. A possible explanation for this might be attempts to increase intake of PMR to compensate the lack of nutrients that can be reflected in more ruminating frequency and drinking water as calves aged. Prior studies indicated the effect of amount of milk, weaning age and method on solid feed consumption and consequently on the development of rumination behavior [65, 66].

During this study, no calves died and no health concerns greater than the minor diarrhea at an early age were reported. Mean rectal temperature of calves was 38.95 ± 0.43 °C, which is above the normal body temperature (38.5 °C) of calves under thermoneutral conditions [67], indicating that these calves may have had difficulty in body thermoregulation. Previously, it has been reported that intensive liquid feeding programs were related to negative effects on calf health, especially on fecal scores in thermoneutral [68] or heat stressed calves [3]. However, in our experiment, calves’ health status was similar between the two planes of milk feeding. On the other hand, increasing milk TS concentration resulted in higher fecal score and diarrhea occurrence from d 4 to 18 of age only when calves received HPM but not LPM. During this period, calves received the same amounts of TS from whole milk but different amount of TS from MR as follows: 17%-HPM > 17%-LPM > 12%-HPM > 12%-LPM with means 13.6 kg (9.6 milk + 4 MR), 12.75 kg (9 milk + 3.75 MR), 9.6 kg (milk) and 9 kg (milk), respectively. In other words, our results showed that HPM-12% TS calves compared to those fed LPM-17% TS received 3.75 kg lower TS of MR and had improved health status as evidenced with lower diarrhea occurrence and fecal score during this age. These results indicate that increasing milk volume might be a better route for providing more milk TS concentration for calves only during early life. Moreover, increasing milk TS concentration resulted in an increase in electrolyte usage and diarrhea medication, but this response was more pronounced in calves fed HPM. Previous studies showed that, compared with Holstein whole milk, MR often provides higher levels of minerals and lactose fraction that can lead to high osmolality (300 vs. ~ 400 mOsm/kg, respectively; [69, 70]), however, mixing errors and feeding practice (mixing MR powder directly into whole milk) may increase the osmolality of the liquid feed (above 600 mOsm/kg) which could exceed the absorptive capacity and possibly lead to osmotic diarrhea [71]. Furthermore, milk feeding volume in each meal and liquid feed osmolality are the most predominant extrinsic factors controlling abomasal emptying, resulting in impairment of gastrointestinal function [72]. According to the higher rectal temperature during times of higher fecal score in calves fed milk with higher TS concentration due to addition of MR, we speculate that the increase in fecal score might be related to non-infectious factors such as higher osmolality of the liquid feed, which may affect water absorption in the intestines [25, 43]. These results may be explained partly by pathogens at an earlier age causing the greater fecal scores accompanied by diarrhea [25, 71], which can be intensified by higher osmolality of the liquid feed and hot weather conditions. Rivas et al. [3] reported that greater fecal score and more diarrhea occurrence may be due to a disturbance of digestion in calves fed large quantities of MR during the first 4-weeks of life in the summer. However, other studies reported no difference in diarrhea occurrence in calves fed higher levels of milk or MR compared with restricted fed calves [13, 21, 61]. These rather contradictory results may be due to differences in method and quality of milk or MR intake, fecal scoring systems, weather conditions, and management factors such as colostrum intake, poor sanitary and housing conditions among studies [13, 40, 61].

## Conclusion

The results of the current study indicated that calves fed HPM-17% TS had greater BW and total ME intake than those fed a LPM-12% during summer. Increasing TS and PM reduced PMR intake without altering rumen function. The research also showed that offering HPM compared to LPM and milk with 17% TS compared to 12% TS had more benefits on feed efficiency and improved skeletal growth of dairy calves during the study. In addition, these results demonstrated that calves that received HPM-12% TS had improved health status compared to those received LPM-17% TS, despite their equal milk TS intake. This indicates that route of milk TS delivery programs may differently affect health of calves during early life. Taken together, these findings suggest that providing a greater amount of milk TS in late-weaned calves has beneficial effects on welfare and growth performance that compensate negative effects of hot weather conditions on PMR intake during summer. A further study could perform cost-effectiveness analysis and assess the long-term effects of feeding a large amount of TS on productive performance for calves reared during summer.

## Data Availability

The datasets used and/or analysed in the current study are available from the authors on reasonable request.

## Abbreviations

PM: Plane of milk
HPM: High plane of milk
LPM: Low plane of milk
MR: Milk replacer
PMR: Partial mixed ration
TS: Total solids
DM: Dry matter
DMI: Dry matter intake
CP: Crude protein
EE: Ether extract
ADF: Acid detergent fiber
NDF: Neutral detergent fiber
NFC: Non fiber carbohydrate
ME: Metabolizable energy
BW: Body weight
ADG: Average daily gain
CNCPS: Cornell net carbohydrate and protein system
NRC: National Research Council
AOAC: Association of Official Analytical Chemists Official methods of analysis
THI: Temperature-humidity index
VFA: Volatile fatty acids
SAS: Statistical analysis system
NNOB: Non-nutritive oral behaviors
